# Editorial: Physiological phenotyping in respiratory diseases: New approaches

**DOI:** 10.3389/fphys.2023.1098839

**Published:** 2023-02-09

**Authors:** G. G. King, F. Formenti, N. Petousi

**Affiliations:** ^1^ Department of Respiratory Medicine, Royal North Shore Hospital, The Woolcock Institute of Medical Research, University of Sydney, Sydney, NSW, Australia; ^2^ Centre for Human and Applied Physiological Sciences, King’s College, London, United Kingdom; ^3^ NIHR Oxford Biomedical Research Centre and Respiratory Medicine Unit, Nuffield Department of Medicine, University of Oxford, Oxford, United Kingdom

**Keywords:** respiratory physiology, lung function, phenotyping, small airways, chronic airways diseases

Airways diseases account for most of the burden of respiratory diseases. In Australia, the burden of chronic respiratory diseases is the 3rd highest behind mental and musculoskeletal diseases. Despite the contrasting pathophysiologic mechanisms underlying asthma, COPD, and bronchiolitis, they all have abnormal structure and function of the peripheral (or small) airways. Peripheral airway dysfunction is fundamental to early COPD and is a strong determinant of symptoms, exacerbations and the development of irreversible airflow obstruction in asthma (Hogg et al., 2004; Burgel, 2011). Bronchiolitis following lung transplantation is purely a disease of the peripheral airways. Despite the obvious importance and clinical relevance of peripheral airway function, it has only been able to be measured meaningfully in the last decade thanks to advances in technology that have enabled sophisticated testing methods to be freely available (Petousi et al., 2019; Usmani et al., 2021).

Thus, it is now time to make the “leap forward” in managing airways diseases, and to go beyond inhaled corticosteroids and long-acting bronchodilators. Asthma and COPD are not singular diagnoses and as such their management necessitates a personalised medicine approach. This approach has been embraced in most medical specialties that facilitate better assessment of a patient’s disease processes, allowing logical management and improved outcomes. Together with inflammatory phenotyping, physiological phenotyping must now be considered. The tools are accessible and the clinical significance of measurements has increasingly been acknowledged. Comprehensive phenotyping is critical if new treatments beyond inhaled therapy are to be realised, as stated emphatically by the Lancet Commissioners on COPD (Stolz et al., 2022).

The papers in this series demonstrate how physiological information complements spirometry, lung volumes and diffusing capacity measurements, and inflammometry. Exhaled gas washout, imaging and oscillometry as measures of lung function have been very well studied. Although oscillometry is somewhat daunting to understand initially, users should soon appreciate the fact that it is a tidal volume breathing test that is sensitive to widespread and heterogeneous peripheral airway narrowing and closure. Ynuk Bossé (2022) describes the underlying principles of oscillometry and how it can be adapted to measure volume-dependent changes, i.e. closing volumes and airway distensibility, that will provide further phenotypic information in airways diseases. Fu et al. demonstrate the use of oscillometry in lung transplant recipients to detect allograft dysfunction earlier, a common complication in which spirometry is very insensitive to leading to early intervention opportunities being missed.

Inert gas washouts measure ventilation distribution to determine primarily, heterogeneity of ventilation, a fundamental characteristic of airways diseases. An even more sophisticated inert gas washout method using a novel technology, described by Smith et al. indicates how distributions of regional lung compliance, deadspace and blood flow can be derived with the use of computational modelling. These physiological indices have not been able to be measured non-invasively before, but may be particularly useful in characterising early stages of disease. Early detection is a hot topic as described in the recent Lancet Commission for COPD (Stolz et al., 2022). The Commissioners make a strong case that COPD should be re-defined to include other physiologic measures, specifically measures of ventilation heterogeneity to promote earlier detection and treatment of COPD.

There are complex mechanisms that underly airways diseases which therefore require more than non-invasive studies of physiology and inflammation. Imaging is another important modality for studying airways diseases, as described by Rutting et al. in explaining the pathophysiology of irreversible airflow obstruction in asthma. They propose that airflow obstruction is not due to airway disease alone and that involvement of the lung parenchyma plays a major role. Imaging provides both structural and functional information, and there are several imaging methods to quantify ventilation 3-dimensionally. Imaging adds topographical information and in doing so, it may allow novel cellular mechanisms involved in lung and airway injury to be more effectively studied *in vivo*. Sallé-Lefort et al. describe the role of Metastasis-Associated Lung Adenocarcinoma Transcript 1 in hypoxia induced lung injury and airway hyperresponsiveness. Sampling both worst and least affected regions of the lung located by imaging, given that disease processes are distributed heterogeneously, may provide novel insights into disease mechanisms and support the role targeted treatment.

Finally, Wu et al. demonstrate how the contribution of reflux to cough can be comprehensively assessed using a number of physiologic and imaging methods. This allows a logical approach to treatment rather than the empirical approach most commonly used presently.

We suggest that an integrated approach that makes use of information from a number of investigative modalities, including physiology, is the current state-of-the-art in respiratory science and clinical medicine. The papers in this series on physiological phenotyping in respiratory diseases, is to accelerate its adoption.
